# Benzyl alcohol improves Ang II-induced vascular and renal injury

**DOI:** 10.55730/1300-0144.5995

**Published:** 2025-02-19

**Authors:** Zhenyu GU, Qi HUA

**Affiliations:** Department of Cardiology, Affiliated Children’s Hospital of Jiangnan University (Wuxi Children’s Hospital), Wuxi, Jiangsu, China

**Keywords:** Pediatric hypertension, benzyl alcohol, blood vessels, kidney injury

## Abstract

**Background/aim:**

The etiology of hypertension in pediatric populations is complex and multifactorial, with metabolic abnormalities playing a fundamental role in the pathogenesis of the condition. This study investigates the therapeutic effects of Benzyl alcohol (BA), identified through metabolomics analysis of pediatric hypertension serum, on Angiotensin II (Ang II)-induced vascular and renal injury in murine models.

**Materials and methods:**

Male C57BL/6 mice were used to establish a vascular remodeling model by continuous 4-week Ang II infusion using a subcutaneous osmotic pump. Bioinformatics was used to identify target metabolites. The tail artery, common carotid artery diastolic, and systolic pressures in mice were determined with a blood pressure monitor. Vascular structure changes were assessed with HE and Masson staining, while kidney pathology was examined using HE. Serum urea nitrogen, creatinine, and cystatin C levels were measured with ELISA kit.

**Results:**

Metabolomics analysis identified metabolite BA as a potential target for hypertension management. Compared to the Ang II group, BA reduced systolic blood pressure by 11.58% and diastolic blood pressure by 14.62% in the fourth week. After sodium nitroprusside treatment, the Ang II group showed reduced vasodilation reactivity versus the control. BA significantly restored this reactivity, unlike acetylcholine. Furthermore, BA was observed to attenuate Ang II-induced vascular mediator thickening, the mediator-to-lumen ratio, and collagen deposition. Ang II administration resulted in renal structural damage and increased concentrations of urea nitrogen, creatinine, and serum cystatin C, which was reversed by BA treatment.

**Conclusion:**

BA exhibits potential in enhancing the vasodilatory response, vascular remodeling, and renal injury associated with Ang II.

## 1. Introduction

Hypertension constitutes a significant risk factor for cardiovascular disease across all age groups, including adults, adolescents, and children [1, 2]. A comprehensive meta-analysis examining global hypertension prevalence in children revealed a combined incidence rate of 4.0%. Notably, the incidence rate escalated from 1.3% during 1990–1999 to 6.0% in the period 2010–2014 [3]. Pediatric hypertension is primarily categorized into two types: primary and secondary, with distinct classification characteristics contingent upon the age of the child. Specifically, children under the age of 10 predominantly exhibit secondary hypertension, whereas adolescents are more likely to develop primary hypertension [4]. The etiology of hypertension in pediatric populations is multifaceted, encompassing genetic predispositions linked to familial history, elevated body mass index resulting from unhealthy lifestyle practices, organ damage incurred during developmental stages, aberrant vascular development, and disorders of the endocrine system [5]. These factors collectively influence the onset and progression of hypertension. For children diagnosed with hypertension, early identification and management of underlying causes, effective blood pressure regulation, prevention of associated complications, and comprehensive investigation into the pathophysiological mechanisms are essential. These efforts provide a critical theoretical foundation for intervention strategies.

Metabolomics represents a burgeoning high-throughput sequencing technology capable of comprehensively characterizing metabolites or matrices within samples, encompassing amino acids, fatty acids, carbohydrates, and other compounds generated by metabolic processes in bodily fluids, cells, and tissues. The metabolites identified as significantly differential are frequently utilized in the screening of biomarkers for disease diagnosis and prediction [6]. Ongoing research into pediatric hypertension has identified abnormal metabolic levels as a major contributing factor to its onset and progression [7]. In pediatric hypertension linked to abnormal cardiovascular development, significant changes in the metabolic profile of the blood are observed, indicating the potential presence of metabolic disorders in this pathological condition. Through the analysis of expression levels and disease risk, it is possible to identify potential prognostic markers and novel therapeutic targets.

Numerous studies have utilized untargeted metabolomic methodologies to preliminarily identify metabolites potentially associated with childhood hypertension, with a particular emphasis on abnormal metabolites including glycerol, phospholipids, amino acids, acylcarnitines, and purines. Moreover, a significant correlation has been identified between changes in the serum metabolic profile of adolescent hypertensive patients and the molecular mechanisms that underpin hypertension [8–10]. Sun et al. employed untargeted metabolomics detection techniques to examine the metabolic profiles of serum samples from children with normotensive and hypertensive conditions, aiming to identify potential metabolic biomarkers. The significant differential metabolites identified through an extensive screening of both positive and negative ions were predominantly associated with amino acid metabolism and glycerophospholipid metabolism [11]. Utilizing the metabolomics detection data from the aforementioned literature, we conducted a reanalysis and identified benzyl alcohol (BA) as a potential target metabolite, guided by the principles of significant differential levels and statistical significance. Subsequently, we investigated its mechanistic role in the progression of hypertension through a series of in vivo and in vitro functional experiments.

## 2. Materials and methods

### 2.1. Data acquisition

Metabolomic data for reanalysis were obtained from the supplementary materials of a published article by Sun et al. [11], and significant differential metabolites were selected based on the principle of variable importance in the projection (VIP) >1.0 and p < 0.05.

### 2.2. Construction of Ang II-induced hypertension mouse model

Animal experiments were approved by the Ethics Committee of Wuxi Children’s Hospital (WXCH2024-01-061). Male C57BL/6J mice, 4 weeks old, were randomly divided into three groups: vehicle, Ang II (ApexBio, A1042, USA), and Ang II+BA, with 5 mice per group. Ang II group: continuous infusion of Ang II (1000 ng/kg/min) for 4 weeks by a subcutaneous osmotic pump; vehicle group: continuous infusion of physiological saline. BA (MCE, HY-B0892, USA) was administered orally once daily (300 mg/kg) along with Ang II infusion, also for 4 weeks. The dose of Ang II [12–14] and BA [15–17], and the duration time was referring to the previously published articles combing verified pathological outcome. In the matter of infusion method, it is performed in accordance with the following operation procedure: (1) Premodeling preparation: measure the mice blood pressure and prepare the necessary number of Alzet model 2006 osmotic pumps, AngII, and saline. Administer Ang II at 1000 ng/kg/min, with each pump containing 250 μL. Incubate AngII and saline at 37 °C for at least 60 h to stabilize drug release. (2) Model surgery: Anesthetize mice with isoflurane, position them prone, and shave the neck and back. Prepare the skin with depilatory cream and iodine. Use sterilized tools to cut a small incision near the shoulder, separate the skin from the fascia, and place the osmotic pump subcutaneously on the back, with the opening facing the tail. Suture the incision (3) Data collection involves continuously operating the osmotic pump for 28 days while monitoring and recording the mice blood pressure and survival status. After 28 days, euthanize the mice with an overdose of pentobarbital sodium and collect materials for future experiments.

### 2.3. Mouse blood pressure measurement

Mouse tail artery pressure was measured weekly using a small animal tail blood pressure monitor. After a week of adaptation, subsequent measurements were taken. The operator should practice multiple times to ensure stable measurements before recording data. Mice were placed in a mouse bag, fitted with a sensor sleeve according to body weight, and measured for systolic blood pressure (SBP) and diastolic blood pressure (DBP) in a 37 °C incubator until stable blood pressure values were obtained. After 4 weeks, mice were sacrificed, and tissues were collected for further experiments.

### 2.4. Vascular tension measurement

The experimental node, prior to the management of vascular tension, represents the terminal time point at which mice are euthanized and vascular tissue is harvested for subsequent tension measurement analysis. This occurs following the administration of various treatments across three distinct groups and varying duration. The relaxation response of mouse aortas was determined using an ex vivo vascular tension meter. Briefly, mice were euthanized by cervical dislocation. Swiftly open the abdominal cavity, extract the thoracic aorta, and place it in 4 °C prechilled, mixed gas-saturated Krebs-Henseleit solution. Aortas were isolated under a dissecting microscope, stripped of surrounding fat, and cut into 2 to 3 mm segments, fixed in a measurement chamber with 37 °C Krebs buffer, and incubated in a 95% O_2_, 5% CO_2_ gas environment for over 30 min. Norepinephrine (NE, MCE, HY-13715, USA) was used to induce vasoconstriction, acetylcholine (Ach, MCE, HY-B0282, USA) for endothelium-dependent vasorelaxation, and sodium nitroprusside (SNP, MCE, HY-B0564, USA) for nonendothelium-dependent vasorelaxation. Attach thoracic aortic strips to needle-like racks in a myograph bath, or thread them with 40 μm wires secured by clamps. Allow the rings to equilibrate for 30 min, set a baseline tension (15 mN), and refresh the solution every 15 min while maintaining this tension for 60 min. Induce contraction with 60 mmol/L KCl for 15 min, then wash with K-H solution until tension normalizes. A final concentration of 10^−4 M NE solution was added to the Krebs buffer in the measurement chamber to maintain vasoconstriction. Ach or SNP was then added in gradient concentrations, with each concentration maintained for over 3 min to induce vasorelaxation, and relevant values were recorded to calculate the relaxation rate.

### 2.5. Tissue staining

Aortas were dissected from the mice, small segments were fixed in 4% paraformaldehyde at room temperature for 24 h. The samples were dehydrated, embedded in paraffin, and cut into 4 μm sections. Sections were stained with hematoxylin-eosin and Masson’s trichrome staining kit according to the manufacturer’s instructions.

### 2.6. Renal function assessment

Renal function was assessed by measuring blood urea nitrogen (BUN) and creatinine (Cr) levels in serum obtained from the abdominal aorta of mice, utilizing an automatic analyzer (Beckman, AU 5800, USA). Additionally, serum cystatin C (CysC) levels were quantified using a mouse-specific ELISA kit (Elabscience Biotechnology, E-EL-M3024, China). Specifically, we obtained the blood according to following steps: Anesthetize the mouse until unresponsive and position it supine on a surgical platform with a test tube under its back. Disinfect the abdomen with 75% isopropyl alcohol and make a midline incision to expose the vessels. Isolate the aorta by removing surrounding fat and cleaning with a cotton ball. Use a 1 mL syringe to draw blood from the aorta, securing with a hemostat, and apply a cotton ball before swiftly withdrawing the needle. Let the blood clot in an anticoagulant tube at room temperature, then separate the serum by inverting and transferring it to a clean tube.

### 2.7. Data statistics

SPSS20.0 was used for statistical analysis, and Graphpad Prism9.0 was used for graphing. One-way ANOVA was used for multi-group statistical analysis, with p < 0.05 indicating statistically significant differences.

## 3. Results

### 3.1. Metabolic profiling analysis and target metabolite screening

**Figure 1:**
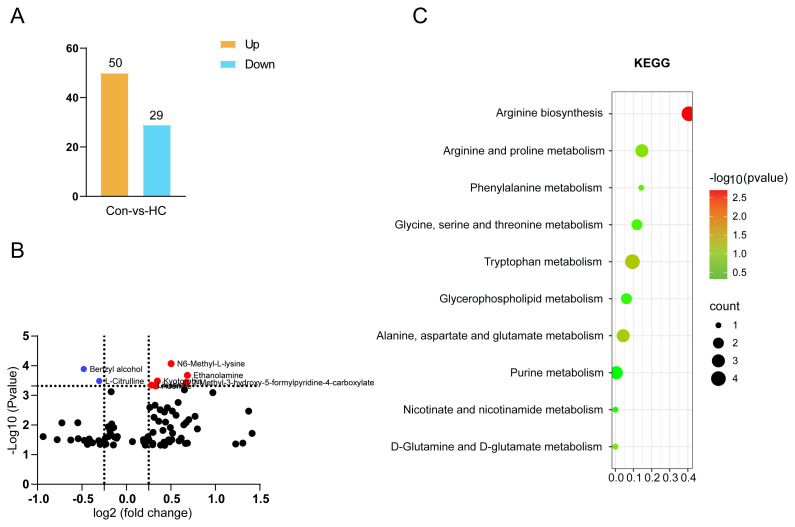
Metabolic profiling analysis and target metabolite selection. (A) Bar chart of the number of upregulated and downregulated differential metabolites; (B) Volcano plot highlighting the identification of upregulated and downregulated metabolites based on threshold values; (C) KEGG enrichment pathway analysis of differential metabolites.

Extract metabolites exhibiting differential expression levels in the hypertensive pediatric cohort from the referenced studies. Reconstruct bar charts and volcano plots to delineate the comprehensive expression profile. Assess the metabolic level disparities between healthy controls (Control, Con) and hypertensive children (HC), and identify target metabolites for further functional validation. In summary, relative to the control group, hypertensive pediatric patients exhibited 79 differentially expressed metabolites, comprising 50 upregulated and 29 downregulated metabolites (Figure 1A). The volcano plot (Figure 1B) illustrated the comprehensive alterations in metabolite levels between the two groups, with red indicating upregulated metabolites and blue indicating downregulated metabolites, defined by a VIP score greater than 1.0 and a q-value less than 0.05. The KEGG pathway analysis identified arginine biosynthesis and tryptophan metabolism as the most significantly enriched pathways (Figure 1C). Benzyl alcohol (BA) was selected as the target metabolite for subsequent functional experiments, based on its high differential expression and statistical significance.

### 3.2. BA alleviates AngII-induced hypertension and vascular injury

**Figure 2:**
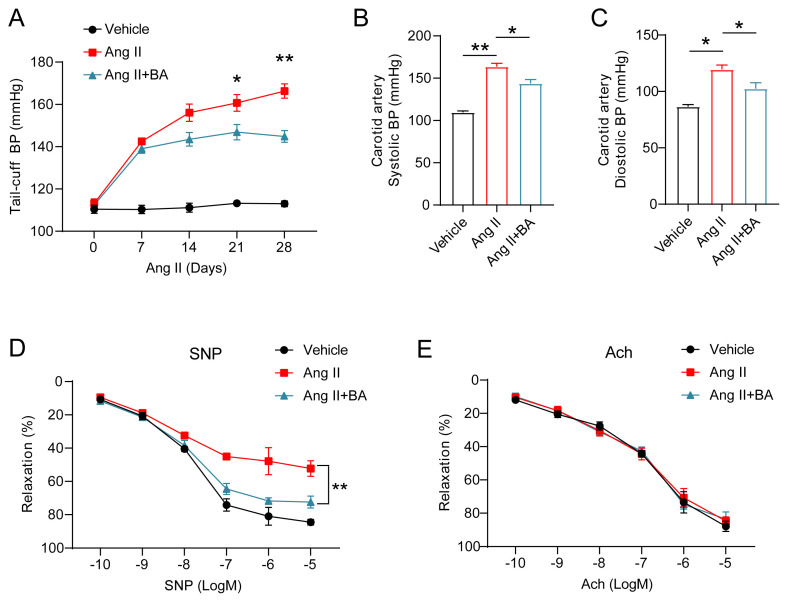
BA alleviates Ang II-induced hypertension and vascular injury. (A) Mouse tail artery pressure measured every 7 days in each group; (B) Systolic blood pressure and (C) Diastolic blood pressure detected in the three groups of mice after 4 weeks of Ang II or physiological saline infusion. Statistical analysis performed under (D) sodium nitroprusside (SNP) and (E) acetylcholine (ACh) stimulation with a concentration gradient. ANOVA, *p < 0.05, **p < 0.01.

As illustrated in Figure 2A, administration of Ang II resulted in an elevation of tail artery pressure in mice when compared to the physiological saline control group. However, treatment with BA markedly attenuated the Ang II-induced increase in tail artery pressure. Furthermore, as depicted in Figures 2B and 2C, BA treatment led to a significant reduction in both systolic blood pressure (SBP) and diastolic blood pressure (DBP) by 11.58% and 14.62%, respectively, at the fourth-week measurement, relative to the Ang II group. Subsequently, the aortic vascular tension across the three experimental groups was assessed. The findings indicated a significant reduction in the vasorelaxation response in the Ang II group compared to the control group. Notably, treatment with BA markedly restored the vasorelaxation response (Figure 2D). Conversely, no significant alterations in vascular tension were observed in either the Ang II group or the Ang II+BA group following Ach treatment (Figure 2E). These results suggest that BA enhances the vasorelaxation response by modulating the function of vascular smooth muscle in the context of hypertension.

### 3.3. BA restores Ang II-induced vascular remodeling

**Figure 3:**
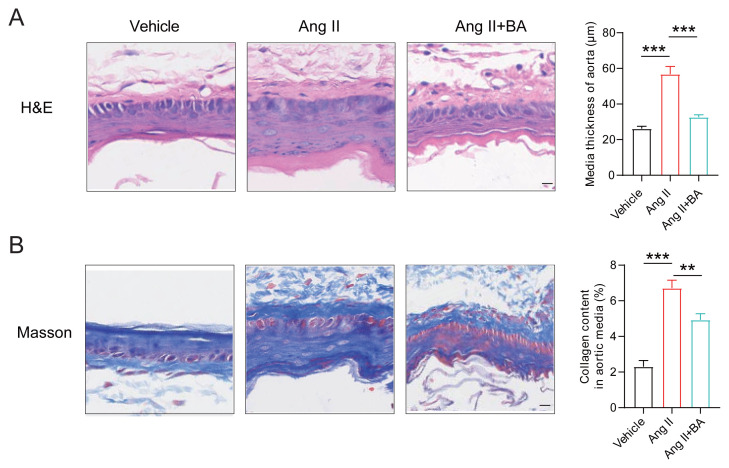
BA improves Ang II-induced vascular remodeling. (A) H&E staining of aortic sections from different groups of mice to analyze medial thickness; (B) Masson staining of peripheral blood from each group of mice to display and calculate collagen deposition. Scale bar = 50 μm. ANOVA, **p < 0.0, ***p < 0.001.

To assess the impact of BA on vascular remodeling prompted by Ang II stimulation, histological analyses using Hematoxylin and Eosin (H&E) staining and Masson’s trichrome staining were conducted to quantify the intimal thickness and collagen expression levels in the aorta. The results from H&E staining indicated that following a 4-week infusion of Ang II, there was a significant increase in the thickness of the vascular media. However, the administration of BA in conjunction with Ang II notably diminished the media thickness (Figure 3A). Correspondingly, Masson’s trichrome staining demonstrated that BA significantly attenuated collagen deposition induced by Ang II (Figure 3B). The above results indicate that BA can improve Ang II induced vascular remodeling.

### 3.4. BA improves Ang II-induced renal injury

**Figure 4:**
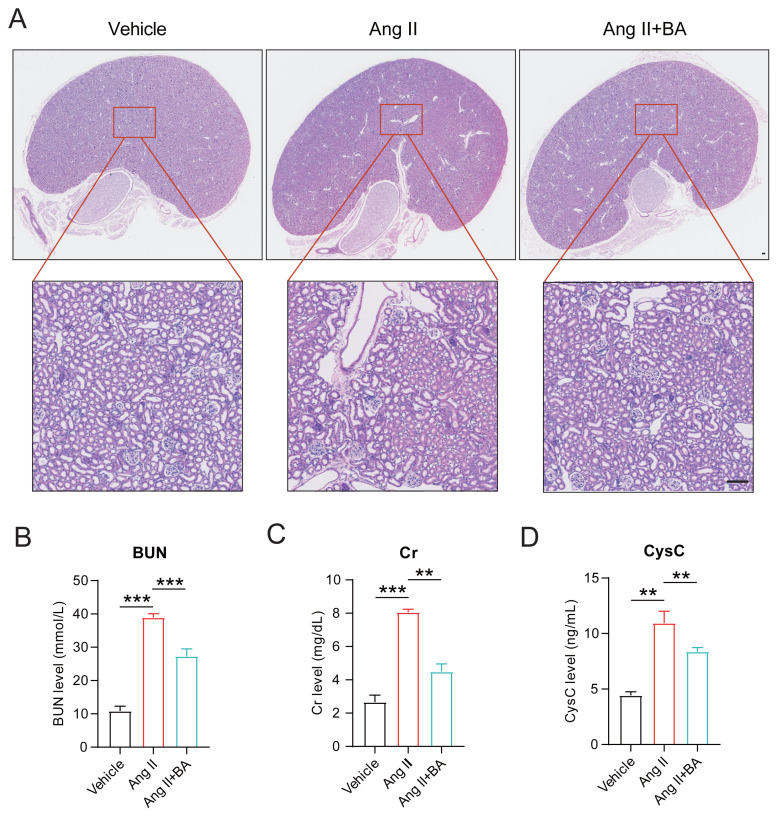
BA improves Ang II-induced renal injury. (A) HE staining results of kidney tissue from different groups of mice and local magnification, scale bar = 100 μm. (B) BUN, (C) Cr, and (D) CysC levels in peripheral blood from each group of mice. ANOVA, **p < 0.0, ***p < 0.001.

Research has demonstrated that prolonged exposure to Ang II can result in renal damage. However, the potential ameliorative effects of BA on renal injury in the context of Ang II-induced hypertension warrant further investigation. In pursuit of this objective, we initially excised the kidneys from mice in the control, Ang II, and Ang II+BA groups, subsequently preparing histological sections for HE staining. The results indicated that the Ang II group exhibited minor glomerular lesions relative to the control group, alongside pronounced renal tubular and interstitial damage, marked renal interstitial edema, and infiltration of inflammatory cells. However, the administration of BA significantly mitigated these renal injury phenotypes (Figure 4A). To further evaluate renal function, we measured the concentrations of blood urea nitrogen (BUN), creatinine (Cr), and cystatin C (CysC) across different experimental groups. As shown in Figure 4B, a significant upregulation of all three biomarkers in the Ang II group was observed. In contrast, combined treatment with BA, the levels of BUN, Cr, and CysC were markedly decreased, suggesting an improvement in compromised renal function.

## 4. Discussion

The etiology of pediatric hypertension has received increasing attention in recent years, and diagnostic and therapeutic technologies targeting it have been significantly improved and refined with the accumulation of clinical experience and in-depth research on molecular mechanisms, bringing effective diagnosis and treatment as well as good prognosis to many children and their families. However, due to some objective reasons, the clinical research results of pediatric hypertension are still hard to reach adults, and there are still problems such as not easy to screen early, single treatment means, and lack of targeted drugs. Therefore, in-depth exploration of its occurrence and development mechanism will lay a theoretical foundation for early screening and the development of drugs for indications. This study is based on the published expression spectrum of hypertensive children to screen and identify significant differentially expressed target metabolites, using a hypertension mouse model, combined with vascular and kidney-related functional experiments, to clarify the role of BA in improving hypertension-induced vascular and kidney injury, providing a new perspective and strategy for the dietary treatment of pediatric hypertension.

Previous studies have shown that the pathogenesis of pediatric hypertension is unclear and complex, involving mitochondrial dysfunction, inflammation, fibrosis, endothelial dysfunction, etc., all of which affect its occurrence and development, among which mitochondrial dysfunction and metabolic disorder-mediated vascular remodeling are considered important causes [18]. Metabolomics, as an emerging biological research method, can characterize metabolic expression spectra through the analysis of datasets, and further reveal the complex biological processes and metabolic pathways involved by using enrichment analysis methods. In recent years, metabolomics research has frequently emerged as a reliable approach for elucidating the pathogenesis of pediatric hypertension and for the development of therapeutic targets [19]. Studies have found that in the pathological environment of hypertension, some amino acid levels are abnormal, such as the downregulation of arginine. Arginine is involved in the synthesis of the efficient vasodilator nitric oxide, and its lower level will directly affect the vasodilator function of the blood vessels [20]; Tryptophan can induce vascular smooth muscle cell contraction, and its level is also different under hypertension induction, leading to abnormal vasodilation and contraction [21]. In addition, young hypertensive patients are found to have higher oxidative stress, lipid metabolism, fatty acid metabolism, and ketone action. In conclusion, metabolomics has the potential to identify aberrant metabolic processes in pediatric hypertension, elucidate the metabolic mechanisms underlying the pathological process, and provide novel insights for the development of personalized diagnostic and therapeutic strategies [22, 23]. However, there are also many drawbacks to the detection results of metabolomics in the hypertensive population. First, metabolites vary greatly in polarity, size, and concentration, and different specimen sources and analysis platforms may produce different results, requiring the joint use of multiple methods to detect and quantify the entire metabolic expression spectrum more scientifically and accurately; second, the level of metabolites varies greatly between individuals and is affected by many factors such as physiology, pathology, emotion, and exercise, requiring the expansion of sample size while strictly controlling the inclusion and exclusion criteria, and further assessment and verification combined with in vivo and in vitro experiments.

We reviewed the literature and analyzed the metabolomic data from Sun et al. [11] on hypertensive and control children to identify key biomarkers that may indicate disease progression or aid in diagnosing pediatric hypertension. Based on the above metabolomic results, this paper screened out the target metabolite benzyl alcohol (BA) with large level differences and high significance. BA is a colorless aromatic alcohol with a mild aromatic odor, which is mostly present in the form of esters in many essential oils in nature. Previous studies have found that BA can improve the synthesis of cAMP by improving the fluidity of renal epithelial cell membranes and can also reduce the activation of inflammatory bodies in a TLR4-dependent manner [15,17]. In addition, BA can improve oxidative stress and mitochondrial dysfunction in liver cells under pathological conditions [16]. These results indicate that BA may enhance endothelial cell function and blood vessel health by improving mitochondrial energy metabolism and reducing inflammation. In the present study, BA was found to mitigate hypertension and vascular damage caused by Ang II in mice. It significantly lowers tail artery pressure, SBP, and DBP by the fourth week compared to Ang II alone. BA also restores vasorelaxation response, which is impaired by Ang II, without affecting vascular tension post-Ach treatment, indicating BA’s potential to improve vascular function in hypertension. Additionally, low BA levels in hypertensive children suggest its potential as a biomarker for early hypertension detection through expanded blood level testing and clinical data analysis. The utilization of BA in clinical interventions, particularly for pediatric hypertension, necessitates further experimental and clinical validation, specifically concerning its concentration, administration methods, and associated toxicological effects to ensure safety. Nonetheless, findings from animal model studies indicate that BA significantly enhances vascular function, suggesting a promising potential for its application.

Studies have confirmed that hypertension is a high-risk factor for diseases such as myocardial infarction, stroke, and kidney disease, among which the relationship between hypertension and kidney injury has been confirmed, and the incidence of nephropathy caused by hypertension is second only to diabetes end-stage kidney disease. The metabolic disorder of the renin-angiotensin-aldosterone system is usually related to hypertension, which can cause an excessive retention of Ang II in the blood vessels [16]. Ang II has been proven to have a strong effect on the kidneys through various mechanisms, including the hemodynamic and nonhemodynamic effects on renal cells, thereby inducing renal fibrosis, inflammatory responses, and oxidative stress [24]. The action of Ang II, as discussed in this paper, significantly induces various renal structural lesions, leading to detrimental effects on kidney function, while the treatment with BA has been shown to improve the pathological phenotype of the kidneys, restoring some degree of normalcy. Furthermore, key indicators of early kidney injury [15], such as BUN, Cr, and CysC, which are critical for assessing renal health, exhibit a notable increase in their levels following prolonged stimulation by Ang II. However, the administration of BA has been found to significantly reduce these elevated indicators, suggesting that BA possesses the potential to ameliorate the kidney injury induced by Ang II, thereby offering a promising therapeutic avenue for mitigating renal damage.

In summary, metabolic abnormalities are considered to be involved in the occurrence and development of pediatric hypertension. Our study shows that the target metabolite BA identified by metabolomic analysis significantly reduces Ang II-induced hypertension and vascular remodeling and improves kidney injury. Our research results provide strong theoretical evidence for the expansion of BA detection in clinical specimens as a biomarker and subsequent treatment of pediatric hypertension as a dietary supplement.
